# Modified Artificial Potential Field for the Path Planning of Aircraft Swarms in Three-Dimensional Environments

**DOI:** 10.3390/s22041558

**Published:** 2022-02-17

**Authors:** Rafael Monteiro Jorge Alves Souza, Gabriela Vieira Lima, Aniel Silva Morais, Luís Cláudio Oliveira-Lopes, Daniel Costa Ramos, Fernando Lessa Tofoli

**Affiliations:** 1 Faculty of Electrical Engineering, Federal University of Uberlandia, Uberlandia 38408-100, Brazil; rafael.mjas@ufu.br (R.M.J.A.S.); gabriela.lima@ufu.br (G.V.L.); aniel@ufu.br (A.S.M.); 2 Faculty of Chemical Engineering, Federal University of Uberlandia, Uberlandia 38408-100, Brazil; lcol@ufu.br; 3 Department of Electrical Engineering, Federal University of Sao Joao del-Rei, Sao Joao del-Rei 36307-352, Brazil; fernandolessa@ufsj.edu.br

**Keywords:** artificial potential fields, Crazyflie 2.0, path planning, quadrotors, vortex field, three-dimensional environments

## Abstract

Path planning techniques are of major importance for the motion of autonomous systems. In addition, the chosen path, safety, and computational burden are essential for ensuring the successful application of such strategies in the presence of obstacles. In this context, this work introduces a modified potential field method that is capable of providing obstacle avoidance, as well as eliminating local minima problems and oscillations in the influence threshold of repulsive fields. A three-dimensional (3D) vortex field is introduced for this purpose so that each robot can choose the best direction of the vortex field rotation automatically and independently according to its position with respect to each object in the workspace. A scenario that addresses swarm flight with sequential cooperation and the pursuit of moving targets in dynamic environments is proposed. Experimental results are presented and thoroughly discussed using a Crazyflie 2.0 aircraft associated with the loco positioning system for state estimation. It is effectively demonstrated that the proposed algorithm can generate feasible paths while taking into account the aforementioned problems in real-time applications.

## 1. Introduction

Path planning consists of defining a sequence of movements from a starting point aiming to reach a desired destination while avoiding collisions with objects in the workspace [1]. Considering the significant advances of robotics and control technologies, this research topic has been extensively studied in the literature in the context of several practical applications. It has become of particular interest to unmanned aerial vehicles (UAVs), which must rely on the accurate and safe movement for accomplishing distinct tasks [2].

Path planning often involves significant complexity owing to constraints associated with the differential speed and acceleration [3], the atmospheric turbulence that makes it difficult to follow a given route accurately [4], the three-dimensional (3D) workspace, and little information about the environment considering limitations of the sensing system [5]. One of the main aspects associated with path planning is the uncertainty with respect to the presence of unknown or unexpected obstacles in the workspace, which demands the continuous monitoring during operation. Therefore, the adaptation and/or recalculation of a given path is essential to ensure the safe operation of vehicles. In the context of global path planning, it requires the previous knowledge of the whole workspace, thus allowing the path to be planned offline, but without the possibility to perform changes in the scenario. On the other hand, online or local path planning relies on the detection of obstacles during the task on a real-time basis [6].

Among the various path planning techniques available in the literature, artificial potential fields (APFs) can be regarded as an interesting local approach for the application developed in this work. The potential fields present a simple algorithmic structure and low computational burden, which makes it feasible for real-time applications [7]. The generated path can maintain a safe distance from obstacles and produce smooth tracking. In addition, this strategy can be promptly applied in dynamic environments with random dimensions since its adaptive algorithm can handle uncertainties in the workspace without requiring any recalculation [8]. However, it still presents inherent drawbacks such as loc al minima trapping, the lack of paths between closely-located objects, unreachable targets, and oscillations in the presence of obstacles or narrow passages [9].

A plethora of algorithms have been proposed in the literature, either based on associating APF with other techniques or by modifying the characteristics of the traditional solution [10]. For instance, in order to minimize problems of local minima and unreachable targets, an improved APF algorithm is presented in [11], which proposes an optimization of the repulsive force orientation angle and the gravitational field function, thus allowing the robot to avoid local minima. Although the results are promising, the author reports that there is a high probability of collision with the target at the final approach instant.

A black-hole potential field was associated with a reinforcement learning algorithm to reduce the occurrence of local minima under multi-target circumstances in [12]. The results showed that a trained agent can adapt to scenarios containing new types of obstacles quickly. However, it is necessary to improve the adaptability of the algorithm because the size of the black hole domain must be defined for different environments. Furthermore, according to the authors, collisions and congestion may occur in multi-agent systems.

In order to mitigate local minima and oscillations in trajectories, an improved APF solution is combined with fuzzy logic in [13]. When the UAV falls into the local minimum position, one or more virtual targets are set to guide the robot out of the dead zone. The association with the fuzzy controller makes the trajectory smoother. However, simulations were performed only considering static obstacles. In environments containing many obstacles, the trajectory may also be excessively longer.

The study proposed in [14] presents a solution for tracking a target in an environment with dynamic obstacles. The authors propose a modification on the repulsive field by taking into account the obstacle dynamics (position and speed) and their dangerousness. The algorithm was tested through simulations in a 3D environment with the presence of a single agent.

Particle swarm optimization (PSO) was combined with the APF method as in [15]. The authors propose a particle swarm-optimized, tangent vector-based APF planning algorithm to shorten the path length and eliminate local minima. Experimental results evidenced that the proposed algorithm could effectively improve path planning efficiency. However, the analysis considered only static environments with a single agent.

The left turning potential field method and virtual target technique are proposed in [16]. The first method forces the UAV to turn 90° to the left and escape the local minimum. The second one sets a virtual target point as an appropriate position when the robot falls into a local minimum trap. Both methods are promising but they have limitations, such as reaching the target in environments with close proximity obstacles.

Rapidly-exploring random trees and APFs are combined into a hybrid strategy presented in [17]. Rapidly-exploring randomized trees are first used to determine a suitable path to maneuver around all obstacles in the workspace. Once a path has been determined, attractive and repulsive potential fields are implemented at all points along it and a gradient optimization algorithm is used to determine paths to reach the desired target. The authors report that further work is necessary to obtain optimal paths since the planned one may be excessively long.

The APF method is combined with rotational vectors aiming at the formation control of UAVs and obstacle avoidance in [18]. The potential fields with rotational vectors around the obstacles adjust the direction of UAV to lead it toward its target without being trapped in local minima. The effectiveness of the algorithm was verified through simulations in the presence of static obstacles only.

An algorithm inspired by the concept of APFs and behavior-based approximation is proposed in [19]. The objective is to allow multiple robots to navigate in formation in an optimal path and time, in addition to avoiding obstacles. The performance of this algorithm is validated in a simulation environment. From the measurements of path length and traveled time, it was observed that the proposed algorithm outperforms techniques such as A*, RRT, and genetic algorithms. However, the analysis was performed solely in the presence of static obstacles.

A hybrid path planning method is proposed in [20] to obtain collision-free paths and improve the vehicle stability by combining the potential field with a sigmoid curve. The repulsive and attractive potential fields are redesigned by considering safety and feasibility issues. The collision avoidance and vehicle dynamics are considered to obtain the shortest path composed of sigmoid curves. The effectiveness is examined by simulations in multi-obstacle dynamic and static scenarios. However, the analysis is performed only for two-dimensional (2D) environments. In addition, the authors report that further studies are necessary to address the local minima problem and to ensure the obstacle avoidance in more complex and unknown environments.

The authors in [21] focus on using a distributed architecture to plan the trajectories of a group of mobile robots. Each robot must be able to detect and avoid collisions with both static and dynamic obstacles present in its environment/neighborhood. A hybrid approach combines three techniques: the APF method, the neighborhood system, and the notion of priority between the robots. The problem of the intersection of robots at the same passage point is solved through the assignment of priority. However, the neighborhood detection technique reduces the influence area of each robot aiming to optimize the calculation time. Additional investigations are required to obtain optimal paths since the planned path and travel time may be excessively long.

An algorithm is introduced in [22] for designing/controlling trajectories of multiple spacecrafts in formation while avoiding collision. The work describes a potential field for formation, which consists of structural and repulsive potentials to allow multiple agents to maintain a regular polygonal shape. Moreover, a rotational potential field in the 3D space is incorporated for collision avoidance with the obstacles. The authors report that the algorithm presents a simple formulation and low computational burden, but the results were evaluated through simulations comprising static obstacles.

In this context, this work presents a path planning algorithm applied to an aircraft swarm in a dynamic environment. Considering that the algorithm simplicity and low computational burden are of major interest in real-time practical applications, a modified APF algorithm is chosen instead of simply combining distinct approaches. Among several solutions available in the literature, it is reasonable to state that combining the rotation and repulsive fields may lead to a more efficient solution for dealing with the inherent limitations in the conventional APF method. In this sense, the main contributions of this work include:

- implementation of a modified APF algorithm with a vortex field that spins over three directions aiming to avoid local minima, collisions, as well as oscillations in narrow passages and in the influence threshold associated with the obstacles;
- development of a technique in which each aircraft analyzes its position in relation to the obstacles and the target, individually determining the best direction of rotation for the vortex field generated by each obstacle aiming at safe motion in the workspace. In this way, agents can adapt to scenarios with new types of obstacles on a real-time basis;
- discussion of experimental tests performed with aircraft model Crazyflie 2.0 in a scenario comprising dynamic targets, as well as obstacles that may require the hierarchical cooperation among agents. From the results, it is possible to verify that the algorithm is viable to be applied in real-time experiments.

The remainder of this article is organized as follows. Section 2 describes the modeling and control of the UAV. Section 3 describes the modified APF approach introduced in this work. Section 4 discusses the methodology, whereas Section 5 presents computational and experimental tests and an in-depth discussion of the obtained results. Section 6 discusses the main conclusions and possible future work.

## 2. Modeling and Control of a Quadrotor

The aircraft model Crazyflie 2.0 was adopted in this work because of its small dimensions, thus allowing the use in narrow indoor environments. Moreover, it has open code firmware and physical interfaces so that it is possible to adapt the aircraft to a wide variety of applications. It is also commercially available, making it easier for other researchers to reproduce the results, and it also allows the focus to be on the development of a robotic application without the need to build a new vehicle, which is outside the scope of this work.

Crazyflie 2.0 weighs about 27 g, with diagonally-opposed axes of 92 mm and a payload of 15 g. The power supply consists of a 3.7 V, 240 mAh lithium polymer (LiPo) battery, which provides a flight autonomy of up to five minutes [23]. The sensing system employs an inertial measurement unit (IMU) that integrates three-axis gyroscopes, accelerometers, magnetometers model MPU-9250, and a pressure sensor model LPS25H. The aircraft also has an ultra-wideband indoor positioning system known as a loco positioning system. It operates through the exchange of high-frequency radio messages between receivers in the aircraft and anchors placed in the environment. This system is based on module DWM1000 and has an accuracy of ±0.10 m [24].

**Figure 1:**
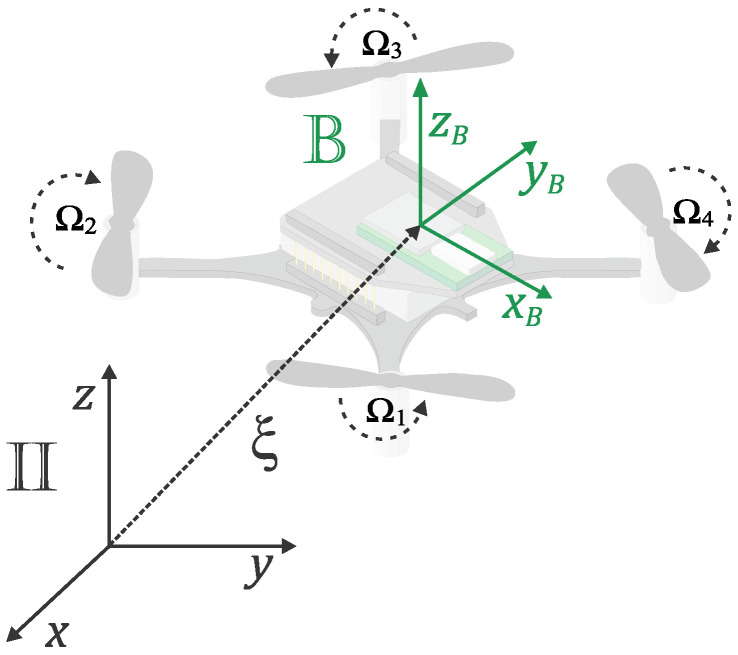
Coordinate systems of a quadrotor.

The dynamic model of the UAV was derived using the Euler–Lagrange representation. For this purpose, one can assume that the aircraft has a rigid and symmetrical structure, with a center of mass that coincides with the origin of the coordinate system fixed to the rigid body. In addition, the propellers are rigid. The thrust and drag forces are proportional to the square of the speeds of the propellers, whose moment of inertia is negligible. Two coordinate systems were employed in the representation: $\mathrm{II}=\left\{\vec{x},\vec{y},\vec{z}\right\}$, which is inertial and fixed to the surface of the Earth; $B=\left\{{\vec{x}}_{B},{\vec{y}}_{B},{\vec{z}}_{B}\right\}$, whose origin coincides with the center of gravity of the aircraft as shown in Figure 1.

Vectors $\xi ={\left[x,y,z\right]}^{T}$ and $\eta ={\left[\phi ,\theta ,\psi \right]}^{T}$ represent the position of the center of mass and the orientation of the UAV, respectively. Additionally, ϕ is the roll angle, θ is the pitch angle, and ψ is the yaw angle, which were obtained using the Tait–Bryan notation. From the general Euler–Lagrange equation, it is possible to write the following equations

(1) $$\begin{array}{l} \left[\begin{array}{l} f_{\xi }\\ \tau_{n} \end{array}\right]=\frac{d}{dt}\left(\frac{\partial L}{\partial {\dot{\lambda }}_{i}}\right)-{\frac{\partial L}{\partial \lambda_{i}}}_{{}_{{}_{{}_{{}_{{}_{{}_{{}_{}}}}}}}}\\ L(q,\dot{q})=E_{c}-E_{p} \end{array}$$

where $f_{\xi }$ and $\tau_{n}$ are the generalized forces and torques, respectively; *L* is the Lagrangian, which is defined as the difference between the kinetic and potential energies; $\lambda =\left[\begin{array}{cc} \xi^{T} & \eta^{T} \end{array}\right]\in {ℜ}^{6}$ is the vector of generalized coordinates.

According to the aforementioned premises, the Lagrangian does not contain energy terms combining the positional and angular derivatives. Therefore, the Euler–Lagrange equations can be divided into two decoupled systems comprising the translational and rotational dynamics.

In the translational system, the Lagrangian depends on the translational kinetic energy and the potential energy according to the following equation

(2) $$L{\left(q,\dot{q}\right)}_{Trans}=E_{cTrans}-E_{p}=\frac{m}{2}{\dot{\xi }}^{T}\dot{\xi }-mgz$$

where *m* is the aircraft mass and *g* is the gravitational acceleration.

Substituting Equation (2) in the Euler–Lagrange formulation (1), as well as considering the force in the inertial coordinate system, the translational dynamics can be defined according to Newton’s second law as

(3) $$\left\{ \begin{array}{l} \ddot{x}=\frac{1}{m}\left[\cos\left(\psi \right)\sin\left(\theta \right)\cos\left(\phi \right)+\sin\left(\psi \right)\sin\left(\phi \right)\right]U_{1}\\ \ddot{y}=\frac{1}{m}\left[\sin\left(\psi \right)\sin\left(\theta \right)\cos\left(\phi \right)-\cos\left(\psi \right)\sin\left(\phi \right)\right]U_{1}\\ \ddot{z}=-g+\frac{1}{m}\left[\cos\left(\theta \right)\cos\left(\phi \right)\right]U_{1} \end{array}\right.$$

where $U_{1}$ is the resulting thrust force produced by the four rotors.

The Lagrangian of the rotational system depends only on the rotational kinetic energy and can be defined as

(4) $$L{\left(q,\dot{q}\right)}_{Rot}=E_{cRot}=\frac{1}{2}{\dot{\eta }}^{T}J\dot{\eta }$$

where J acts as the inertia matrix expressed in terms of the generalized coordinates η.

Similarly, substituting (4) in (1), the angular part of the Euler–Lagrange equation assumes the following form

(5) $$\left\{ \begin{array}{l} \ddot{\phi }=\frac{I_{yy}-I_{zz}}{I_{xx}}\dot{\psi }\dot{\theta }+\frac{\tau_{\phi }}{I_{xx}}\\ \ddot{\theta }=\frac{I_{zz}-I_{xx}}{I_{yy}}\dot{\phi }\dot{\psi }+\frac{\tau_{\theta }}{I_{yy}}\\ \ddot{\psi }=\frac{I_{xx}-I_{yy}}{I_{zz}}\dot{\theta }\dot{\phi }+\frac{\tau_{\psi }}{I_{zz}} \end{array}\right.$$

where $\left[\tau_{\phi }\;\tau_{\theta }\;\tau_{\psi }\right]$ are the torques associated with the unbalanced thrust forces caused by the rotors and $\left[I_{xx}\;I_{yy}\;I_{zz}\right]$ are inertia moments.

It is worth mentioning that one can obtain the dynamic equations of a quadcopter following the procedure described in [25]. Crazyflie 2.0 aircraft parameters are also available in [26]. Furthermore, the parameterized model was used in this work to tune the controllers.

**Figure 2:**
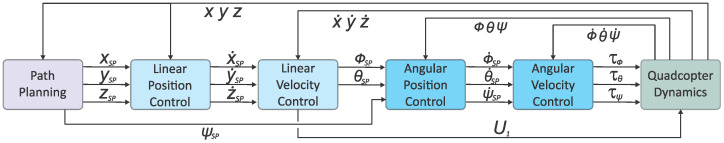
Information flow of the cascade controller.

Since the quadcopter is an underactuated mechanical system, a cascade control strategy was used, in which the inner loop regulates the orientation, whereas the outer loop is responsible for the translational system as shown in Figure 2. Each loop is divided into two subsystems responsible for regulating the zero-order states and their respective derivatives, resulting in a total of 12 controllers.

The proportional–integral–derivative (PID) controller employed in the quadcopter can be represented in terms of the following expression

(6) $$\left\{ \begin{array}{l} P_{k}=K_{p}e_{k}\\ I_{k}=I_{k-1}+K_{i}T_{s}e_{k}\\ D_{k}=K_{d}\frac{e_{k}-e_{k-1}}{T_{s}}\\ u_{k}=P_{k}+I_{k}+D_{k} \end{array}\right.$$

where *K_p_*, *K_i_*, and *K_d_* are the proportional, integral, and derivative gains, respectively; *T_s_* is the sampling period; *e_k_* and *e_k_*_−1_ are the present and past values of the error signal, respectively; *u_k_* is the output signal. Anti-windup limiters are associated with the integral term |*I_lim_*| and the output signal |*u_lim_*|.

Crazyflie 2.0 presents a basic tuning for the controller, which can be improved to meet the specific constraints of this project. The controller was then tuned considering the integral absolute error (IAE) as an evaluation metric. The analysis was performed in a computational environment based on the parametrized model of the aircraft and further experimental validation. The obtained parameters are shown in Table 1. The state estimation of the aircraft is performed by means of the sensory data fusion obtained from the IMU, as well as information from the local positioning system through an extended Kalman Filter [23]. The outer loop operates at 100 Hz. In the inner loop, the angular rate and angular position controllers operate at 500 Hz and 250 Hz, respectively.

## 3. Artificial Potential Field

Artificial potential field was introduced by Oussama Khatib in 1986 and consists of filling the workspace with an artificial scalar potential field. The final position presents an attractive potential, whereas the obstacles act at generating repulsive forces such that the robots are guided toward the final objective, also avoiding obstacles that may exist in the environment [27].

### 3.1. Attractive Field

The attractive potential $\left(U_{at}\right)$ for any point in the workspace is defined through a quadratic function [28]

(7) $$U_{at}\left(q\right)=\frac{1}{2}k_{at}{\left\|q-q_{target}\right\|}^{2}$$

where $k_{at}$ is the attractive field gain coefficient; $q={\left[x,y,z\right]}^{T}$ corresponds to the aircraft position; $q_{target}={\left[x_{a},y_{a},z_{a}\right]}^{T}$ represents the target position.

The function is continuously differentiable and non-negative in the whole domain becoming zero at the destination coordinates. The attraction force $\left(F_{at}\right)$ coming from this field is represented as

(8) $$F_{at}\left(q\right)=-\vec{\nabla }U_{at}\left(q\right)=-k_{at}\left(q-q_{target}\right)$$

### 3.2. Repulsive Field

The repulsive potential $U_{r}$ is intended to create a barrier around every obstacle that exists in the workspace. Furthermore, it should not influence the aircraft motion when it is far enough away [29]. One way to meet these constraints is to define the repulsive potential generated by each obstacle according to the following expression

(9) $$\left\{ \begin{array}{cc} U_{r}\left(q\right)=\frac{1}{2}k_{rep}\left(\frac{1}{\rho \left(q\right)}-\frac{1}{\rho_{0}}\right), & \rho \left(q\right)\leq \rho_{0}\\ 0, & \rho \left(q\right)>\rho_{0} \end{array}\right.$$

where $k_{rep}$ defines the repulsive field gain coefficient; ρ(q) is the distance between the aircraft and the closest point to the obstacle; $\rho_{0}$ is the distance of influence.

The repulsive force $\left(F_{r}\right)$ created by each obstacle can be calculated as

(10) $$\left\{ \begin{array}{cc} F_{r}\left(q\right)=k_{rep}\left(\frac{1}{\rho \left(q\right)}-\frac{1}{\rho_{0}}\right){\left(\frac{1}{\rho \left(q\right)}\right)}^{2}\vec{\nabla }\rho \left(q\right), & \rho \left(q\right)\leq \rho_{0}\\ 0, & \rho \left(q\right)>\rho_{0} \end{array}\right.$$

### 3.3. Vortex Field

In this work, the rotational or vortex fields are implemented to avoid local minima, as well as oscillations in narrow passages and in the threshold influence of obstacles. For this purpose, it is essential that the direction of rotation of the vortex is properly defined, otherwise the technique may not provide good results. In this sense, as one of the main contributions of the present study, the agents are modified so as to define the best direction of the vortex field rotation automatically and independently according to its position with respect to each object in the workspace. It is worth mentioning that in 3D flight spaces, the direction of rotation must be defined in each of the three planes.

**Figure 3:**
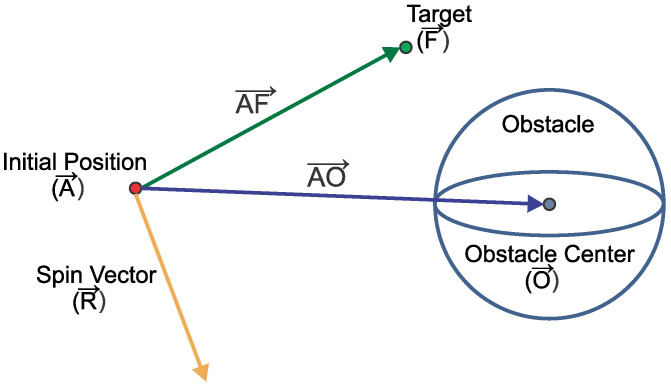
Determining the spin vector of the vortex field.

The vortex field is determined by performing a 3D rotational operation on the repulsive field generated by each obstacle. This is accomplished through three successive rotation operations on each canonical axis. To determine the direction of rotation, first it is necessary to know the spin vector $\vec{R}$ according to (11).This parameter can be calculated from the vector multiplication between $\vec{AF}$, which is defined by the difference between the current position $\vec{A}$ and the final position $\vec{F}$, and vector $\vec{AO}$, which connects the current position to the center of the detected obstacle $\vec{O}$ as shown in Figure 3.

(11) $$\vec{R}=\vec{AF}\times \vec{AO}$$

Once the rotation vector is determined, it can be decomposed into three components $\vec{R^{X}}$, $\vec{R^{Y}}$, and $\vec{R^{Z}}$, from which the rotational fields are calculated according to the operations denoted in Table 2. Additionally, parameter FnmV represents the vortex field force of the *m*-th obstacle acting on the *n*-th agent, whereas FnmVX, FnmVY, and FnmVZ are its respective components. The gain coefficient of the rotational field corresponds to coefficient $k_{V}$.

### 3.4. Cooperation

In this work, a sequential hierarchical cooperation approach between the agents in the workspace is implemented. Thus, each aircraft defines its respective subsequent reference position based on the current and planned states of the aircrafts belonging to the higher hierarchical layers.

Once the new position of the first robot is calculated, it is shared with the other agents. The next vehicle then makes its decision knowing the information obtained from the first one to avoid collisions. The process is repeated sequentially until the last agent. It is important to highlight that each robot is considered as an obstacle by its peers, generating repulsive and vortex fields that influence the movement of other individuals.

### 3.5. Resultant Field and Path

The resultant force on each aircraft corresponds to the combined action of vector fields from the target, obstacles, and other agents. From the resultant field, the algorithm is able to obtain a viable route for each agent using the steepest descent method while also respecting the speed limit of the aircraft. For this purpose, a search vector containing *N* workspace pointing in the direction of the steepest descent of the resultant potential field is obtained. The value of *N* is determined from (12) as a function of the maximum aircraft speed *V_lim_*, the estimated sampling time between the calculation of each route point given by *T_st_*, and the sampling resolution of the workspace corresponding to *Res*. Once the route points are determined for each agent, this very same procedure is repeated iteratively until all the aircrafts are close to their respective targets while also respecting the imposed reference values.

(12) $$N=V_{lim}T_{st}Res$$

## 4. Methodology

The APF algorithm presented in this work was implemented in MATLAB software. For path planning purposes, a scenario involving a swarm with two aircrafts in the presence of a target and a dynamic obstacle was proposed. The scenario was arranged in the form of a challenging task for traditional path planning algorithms. In other words, a single target is defined for both aircrafts in order to assess the mutual repulsion between them and the capability of the algorithm to keep them equally distant from the target. In addition, the target trajectory defined in a ring shape was generated to remain hidden by the obstacle during a few seconds, aiming to evaluate the decisions made by each agent to avoid collisions. The reduced dimensions and short execution time of the tests aims to reproduce the procedure easily for small aircrafts with low flight autonomy in indoor environments. Technical data involving the scenario are available in Table 3.

In order to evaluate the performance of the method proposed in this work, the algorithm is compared with the conventional APF approach, which does not include the vortex field as proposed in [27], and with the left turning APF algorithm (LTAPF) presented in [16].

The conventional APF algorithm does only incorporate attractive and repulsive fields. Therefore, it is not capable of overcoming issues such as local minima and oscillations in trajectories. The LTAPF method employs a vortex field that drives the agent to the left whenever it gets stuck on a local minimum point.

The comparison aims to demonstrate that the use of rotating fields combined with the possibility to determine the best spin direction of the vortex field associated with each agent leads to improved performance while also overcoming inherent problems in the traditional APF method without a significant increase in computational burden.

Computer simulations were carried out using the aforementioned path planning algorithms in the scenario described in Table 3. The metrics for comparing the results include the presence of collisions, the final distance to the target, the total distance covered by each aircraft, and the average distance between each aircraft and the target. The presence of oscillations in the trajectories is also analyzed. The simulations do not take into account the dynamics of the quadrotor but the kinematic constraint only with respect to the translational velocity.

**Figure 4:**
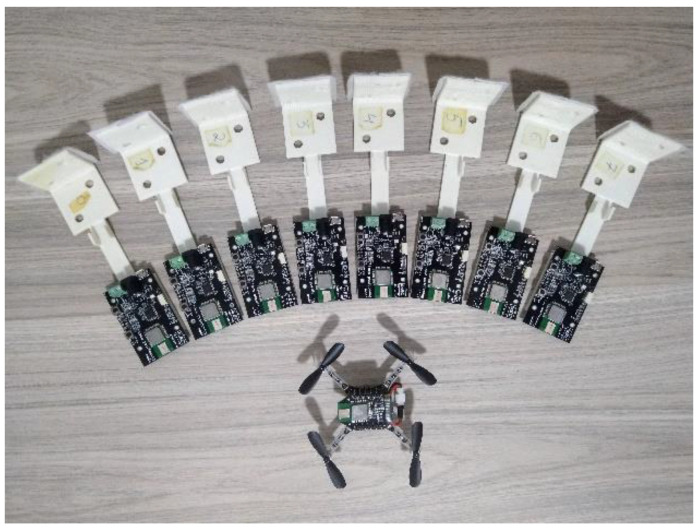
Anchors and quadcopter Crazyflie 2.0.

After analyzing the computational results, experimental tests were carried out with a real aircraft in order to evaluate the feasibility in real-time applications. The experiments used Crazyflie 2.0 quadcopters combined with the indoor positioning system. Figure 4 shows the eight anchors of the positioning system, as well as a receiver (tag) attached to the top of the aircraft.

The experimental tests were carried out using a Python script written according to Algorithm 1. The code is responsible for allowing the radio communication with the aircrafts, enabling the path generator written in MATLAB (Algorithm 2) and for storing the measured data.

Algorithm 1 operates as follows. Initially, the variable that controls the total test time (*t*) and the initial positions of the aircraft are declared. After the algorithm checks the status of the quadcopters, they are sent to the starting positions. Variable *t*_*test* is initiated in line 7 while counting the time elapsed after each test. Then, the main loop is executed until the test time is reached.

Setpoints are sent at intervals of about 1000 ms, while the current aircraft positions are received at intervals of 100 ms and stored in *UAV*_*position* matrix. This matrix is arranged in such way that the columns represent the sequence of coordinates (*x*, *y*, *z*) of all vehicles sequentially, whereas the rows are constantly updated with the values sent by the quadcopters.
**Algorithm 1: Python Interface****1****Input:** none**2****Output:***UAV_position*, *UAV_SP***3****Declare:***t*, *UAV_initial_position***4***Check_crazyflie* ()**5***Send_crazyflie* (*UAVs_initial_position*)**6***UAV_position* = Get_crazyflie_position ()**7**Start (*t_test*)**8****While** *t_test* <= *t* **do****9**       Start (*t_control*)**10**       *UAV_position_vec.append* (*UAV_position*)**11**       *Time_vec.append* = *t_test***12**       *New_SP* = *Modified_vortex_APF* (*UAV_position* (end), *t_test*) *(presented in Algorithm 2)***13**       *Send_crazyflie* (*New_SP*)**14**       *UAV_SP.append* = *New_SP***15**       Wait (*t_control* => 1000 “ms”)**16**** end While****17*** Land_Crazyflie* ()**18** Save *UAV_position*, *UAV_SP*, *Time*_vec

The MATLAB function, called in line 12, receives the instant positions of all quadrotors and time information so as to handle the virtual scenario properly. After the execution, the new setpoints are sent to the vehicles (line 13) and stored on *UAV*_*SP* matrix (line 14), which has the same structure as *UAV*_*position*.

Once the test exceeds the defined value *t*, the script exits the “while” loop, calls the high-level function to land the quadcopters safely (line 17), and stores the data in txt files so they become accessible for latter analysis and plotting (line 18).

The modified APF method proposed in this work is describe in Algorithm 2. It receives the instant time (*t*_test) and vehicle positions (*UAV_position*) in order to calculate the new setpoints for the quadrotors. First, based on the scenario configuration, the target and initial positions of obstacles are declared, as well as their respective speeds. In addition, the coefficient gains for the attractive (*k_a*), repulsive (*k_r*), and vortex (*k_v*) fields and the velocity constraint for the Crazyflie aircrafts (*max_vel*) are also declared (line 3). This assignment occurs only once and is not callable by the Python script every cycle.

The positions of the target and obstacle are updated. Once *UAV_position* vector contains the coordinates (*x*, *y*, *z*) of each aircraft, the amount of Crazyflie aircrafts is determined as shown in line 7. Then, a “for” loop is executed for calculating the setpoints for each *j*-th vehicle. A vector containing the indexes corresponding to the *j*-th aircraft is then created in line 9, considering that the first index of a vector is 1.

The script creates a cell array namely *Obs_expand*, which comprises the information of the positions of the obstacle and all the other vehicles “*not*(*j*)” (line 10). After that, the spin directions for the obstacle and all the remaining aircrafts are determined, resulting in the later calculation of the attractive, repulsive, and vortex fields.

The workspace resultant field for the *j*-th quadrotor is calculated from the weighted sum of the aforementioned fields (line 15) associated with their respective gains. Thus, the new setpoint is found using the steepest descent method (line 16). Before this procedure is repeated for the next agent, the setpoints are properly stored in *UAV_SP* vector. Once the new setpoints are determined for all vehicles, the routine returns *UAV_SP*.
**Algorithm 2: Modified APF****1****Input:** *UAVs_position*, *t_test***2****Output:** *UAV_SP***3****Declare** *Target*, *target_speed*, *Obstacle*, *obstacle_speed*, *k_a*, *k_r*, *k_v*, *max_vel***5***Target_pos* = update (*Target*, *target_speed*, *t_test*)**6***Obstacle_pos* = update (*Obstacle*, *obstacle_speed*, *t_test*)**7***n_UAV* = length (*UAV_position*)/3**8****for** *j* = 1: *n_UAV* **do****9**       *j_index* = [1 + 3 ∗ (*j* − 1) 3 ∗ *j*]**10**       *Obs_expand* = *(Obstacle_pos*, *UAV_position* (not (*j_index*)))**11**       *spin_dir* = *Vortex_field_spin_direction* (*Target_pos*, *Obs_expand*, *UAV_position* (*j_index*))**12**       *Att_field* = *Attractive_field* (*Target_pos*)**13**       *Rep_field* = *Repulsive_field* (*Obs_expand*)**14**       *Vor_field* = *Vortex_field* (*Obs_expand*)**15**       *WS_field* = *k_a* ∗ *Att_field* + k_r ∗ *Rep_field* + *k_v* ∗ *Vor_field***16**       *new_SP* = *steepest_descent* (*WS_field*, *max_vel*)**17**       *UAV_SP (j_index*) = *New_SP***18**** end for****19**** Return:** *UAV_SP*

The workspace was sampled at a resolution of 50 points per meter. This value was determined during experimental tests and represents a good tradeoff between accuracy and computational burden, comprising a maximum system error of ±0.0144 m. The trajectories are calculated considering that each aircraft is a material point allocated at its center of mass, while the dimensions of the obstacles are expanded by a safety factor equals to 0.2 m.

The safety factor was chosen as the sum of the radius of the aircraft, that is, approximately 0.0661 m and the measurement uncertainty of the local positioning system, provided by the manufacturer, which is equal to 0.1 m, added by a 20% security margin.

The simulations and experimental tests were carried out on a computer with the following configurations: Ubuntu 20.04 LTS operating system, Intel Core I7-4790 3.60 GHz processor, 16 GB of RAM memory, and GTX960 video graphic card.

## 5. Results

### 5.1. Simulation Results

In order to evaluate the performance of the proposed algorithm, the obtained path is compared with those generated by the conventional APF algorithm and LTAPF. Considering the measurement uncertainty in the aircraft positioning, the algorithm recognizes that there is a possible collision between the aircraft and the obstacle when the distance between them is less than the safety factor (0.2 m). Furthermore, a collision between quadcopters is expected to occur if the distance between them is less than twice the safety factor, that is, 0.4 m. The parameters used in the tests with the algorithms are shown in Table 4.

In order to analyze the simulation results, the metrics used for comparison are presented in the Table 5.

**Figure 5:**
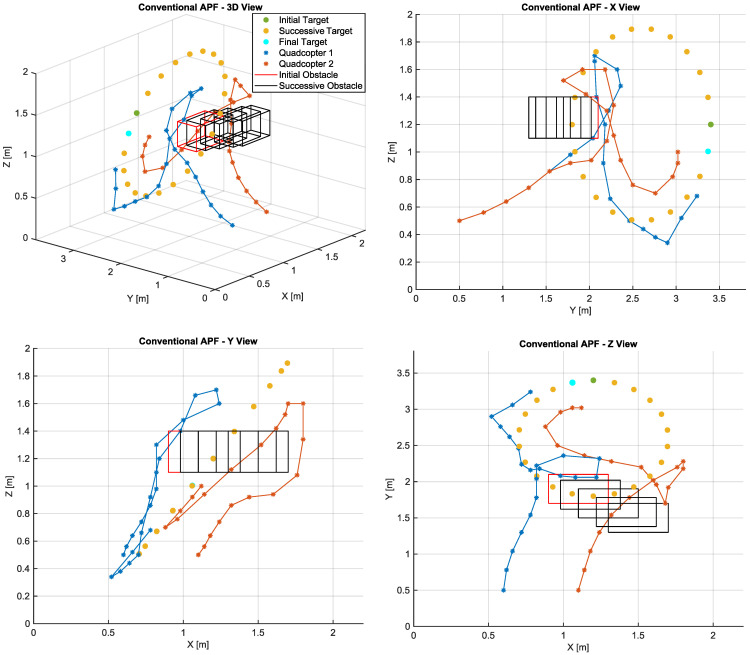
Paths calculated by the conventional APF algorithm.

The path planned by the conventional APF approach is shown in Figure 5. The initial target coordinates are *x* = 1.2 m, *y* = 2.6 m, and z = 1.2 m, represented in the plot by the dark green dot. The target trajectory is marked by successive yellow dots, while the final position is represented in cyan. The obstacle initial coordinate is identified by the red rectangle and its trajectory is represented in black. Quadcopter 1 is identified in blue and its initial position is *x* = 0.6 m, *y* = 0.5 m, and *z* = 0.5 m. In turn, quadcopter 2 is identified in orange, whereas its initial coordinates are *x* = 1.1 m, *y* = 0.5 m, and *z* = 0.5 m. The two aircrafts take off at the same time, aiming to reach the target while dodging the dynamic obstacle. It is noteworthy that UAV1 belongs to a higher hierarchical layer. Therefore, it has priority in route planning. Then, after UAV1 makes its route available to the lower layers, UAV2 defines its own planning. Therefore, the two aircrafts operate in sequential hierarchical cooperation.

Compared with the other methods, this algorithm was able to provide the shortest routes for both UAVs. However, it was not able to avoid collisions with the obstacle. Although there are no local minima, the lack of a turning field to generate a more efficient route allowed UAV1 to collide with the obstacle in the fifth and sixth setpoints (SPs) generated by the algorithm. In turn, UAV2 collides with the obstacle after going around it and heading toward the target in the thirteenth SP. Furthermore, this algorithm provided the worst final target approximation among all evaluated methods.

Videos referring to the computer simulations are available as Supplementary Materials (Videos S1–S3).

**Figure 6:**
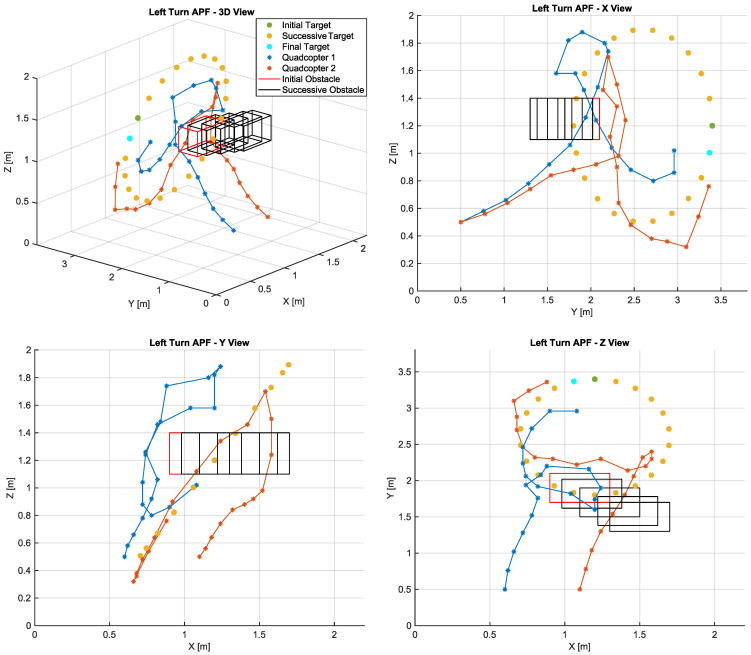
Paths calculated by the LTAPF algorithm.

The LTAPF method calculated the routes presented in Figure 6. The existence of a turning field allows UAV2 to avoid the collision with the obstacle. However, it was not able to avoid the collision with UAV1 in the fifth SP. In comparison with the other methods, the greater distance covered by UAV2 demonstrates that, although the algorithm was successful in avoiding the obstacle, it proposed a longer route. Furthermore, the final approach to the target by both aircrafts is improved compared with the conventional APF method, but it does not outperform the algorithm proposed in this article. It is the authors’ opinion that such behaviors may be due to impossibility of changing the turning field direction.

**Figure 7:**
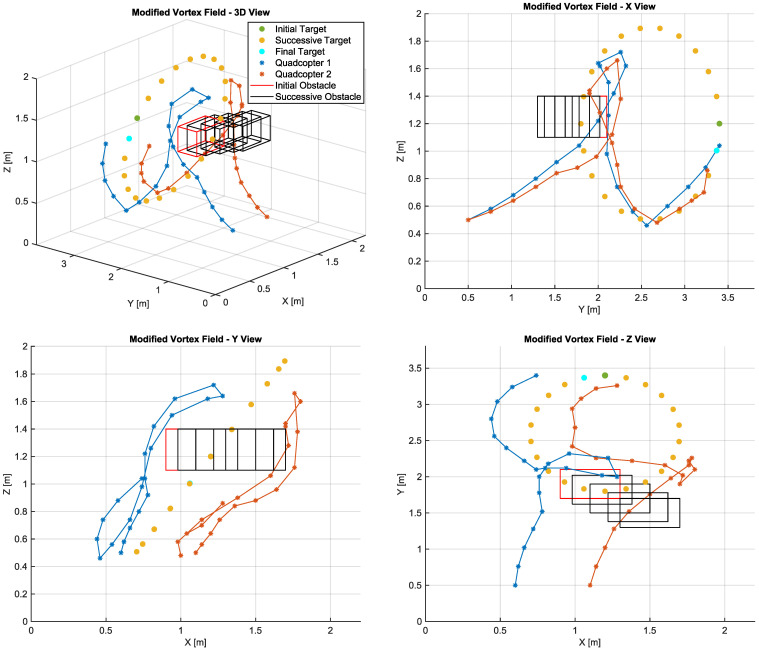
Path calculated by the proposed vortex field APF.

Figure 7 presents the 3D view of the trajectories generated by the proposed method. It is observed that the generated path allows the aircrafts to make detours around the obstacle, evidencing that the vortex field direction can differ between both vehicles when they face a same obstacle. This characteristic provides the vehicles with the capacity to decide the best way to avoid both a local minimum and a poor choice of path, which may be of major importance when it comes to a dynamic obstacle.

Although both aircrafts are supposed to reach the same target, it is observed that the destination of each agent does not correspond exactly to the target position so as to avoid collisions. Compared with the other techniques, the proposed algorithm was capable of providing a closer approximation to the target while keeping a safe distance between the agents.

Regarding the other metrics, it was observed that the path of UAV1 was the longest among the tested algorithms. However, it was the only method that avoided a collision with the obstacle. The total distance covered by UAV2, although longer than that covered by the conventional algorithm, proved to be shorter than the route calculated by LTAPF, being equally capable of avoiding collision. The average distance to the target for UAV1 was the shortest, whereas it was the second shortest for UAV 2, that is, only slightly above that presented by LTAPF. However, it did not show oscillations in the final part of the route.

### 5.2. Experimental Results

**Figure 8:**
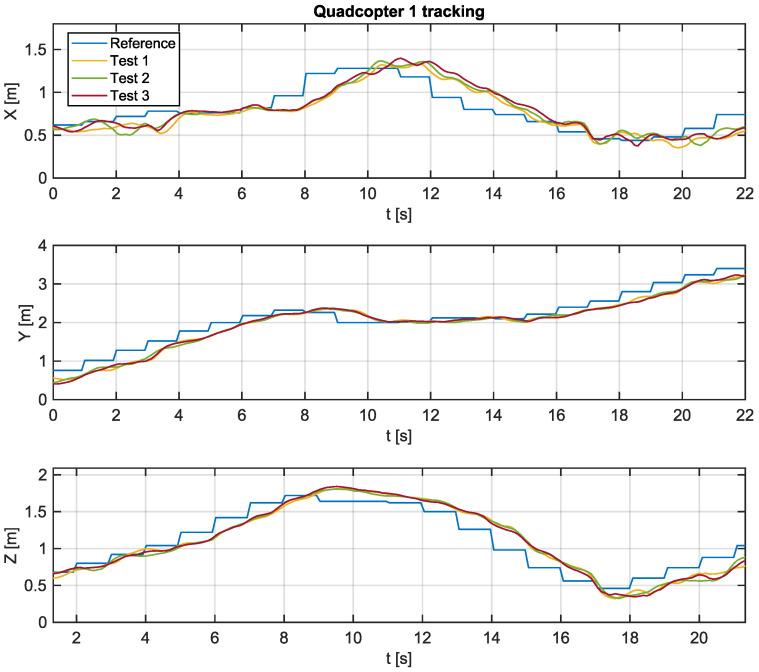
Path tracking of UAV 1.

**Figure 9:**
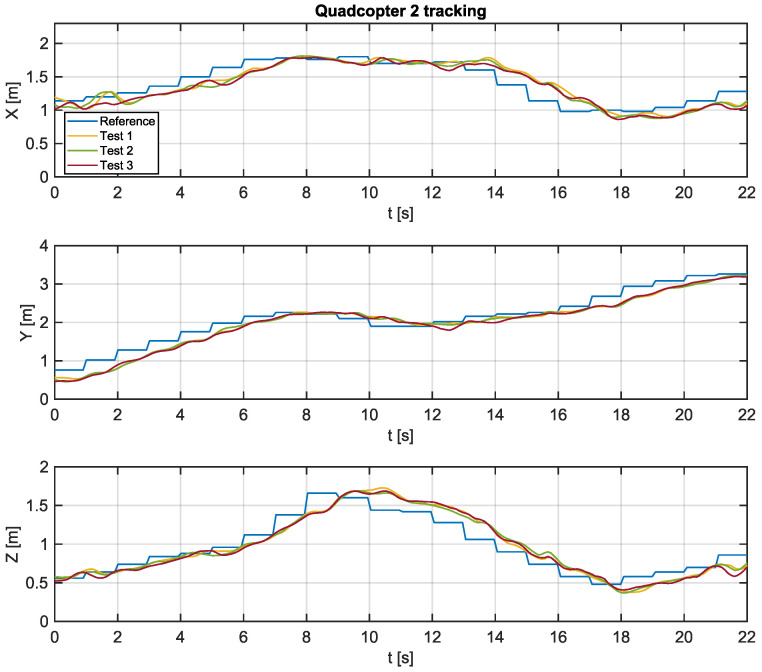
Path tracking of UAV 2.

Experimental tests were carried out using the algorithm proposed in this work in order to verify the feasibility of its implementation in practical real-time applications. The Python script, which communicates with Crazyflie 2.0 aircraft, sends position references at intervals of 1 s, while state estimates coming from the local positioning system are sampled at intervals of 100 ms. Figure 8 and Figure 9 present the trajectories of quadcopters 1 and 2 based on the tracking of the paths generated by the proposed algorithm.

Experimental tests were performed three times to verify possible inconsistencies in tracking the trajectories, possibly caused by interference in radio communication or distortions due to battery discharge. It is possible to verify that the aircrafts presented a similar behavior in all tests.

Overall, it is reasonable to state that the tracking is successful. Moreover, there is a steady-state error for the ramp tracking pattern, even though the error is null when the reference is constant.

Table 6 and Table 7 present a summary of the data collected during the three experiments with the UAV1 and UVA2, respectively.

From the results obtained, it is possible to verify that the aircrafts are capable of tracking the trajectories without collisions during the flight. Furthermore, it is verified that the proposed algorithm presents low computational burden. Therefore, it represents a good alternative for practical real-time applications involving dynamic environments.

## 6. Conclusions

This work has presented the implementation of a modified APF algorithm with a vortex field that spins over three directions in order to overcome the main limitations associated with the traditional APF approach. A strategy was developed in which each aircraft could individually determine the best direction of rotation for the vortex field generated by each obstacle.

The algorithm introduced in this work was analyzed in a scenario involving hierarchical cooperation and the presence of a moving target and obstacle. The results were compared with other similar methods available in the literature. It is reasonable to state that including vortex fields with individually determined directions contributes to reducing the risk of collision with obstacles and between agents. It allows mitigation of the problem of the unreachable targets, as well as reducing path oscillations and the distance traveled by the aircrafts.

Experimental tests were performed to verify the feasibility of the proposed path planning algorithm. From the obtained results, it becomes evident that the technique presents prominent performance for practical applications in real-time 3D scenarios while also avoiding significant loss of performance due to high computational burden.

Future work includes incorporating the vehicle model into the path planning algorithm aiming to obtain smoother routes. In addition, the use of sensors that enable the aircrafts to detect targets and obstacles in the environment are expected to be evaluated. Increasing the number of aircraft and dynamic obstacles in the environment is also of major interest to evaluate the impact on the computational performance of the proposed approach, as well as the adaptability of the algorithm.

## Figures and Tables

**Table 1:** Gains of the PID controller.

Variable	*K_p_*	*K_i_*	*K_d_*	|*I_lim_*|	|*u_lim_*|
*X*	1.8	0.0	0.0	5000	1.10
*Y*	1.8	0.0	0.0	5000	1.10
*Z*	1.8	0.7	0.2	5000	1.10
$\dot{X}$	25.0	5.0	0.0	5000	22.0
$\dot{Y}$	25.0	5.0	0.0	5000	22.0
$\dot{Z}$	25.0	15.0	0.0	5000	32.7
ϕ	8.0	3.0	0.0	20.0	-
θ	8.0	3.0	0.0	20.0	-
ψ	4.0	1.0	0.35	360.0	-
$\dot{\phi }$	250.0	500.0	2.5	33.3	-
$\dot{\theta }$	250.0	500.0	2.5	33.0	-
$\dot{\psi }$	120.0	16.7	0.0	166.7	-

**Table 2:** Definition of the vortex field.

Component	Signal	Mathematical Operations
$\vec{R^{X}}$	≤0	FnmVX(q)=FnmVX(q) FnmVY(q)=FnmVY(q)+KV⋅FnmVZ(q) FnmVZ(q)=FnmVZ(q)−KV⋅FnmVY(q)
	>0	FnmVX(q)=FnmVX(q) FnmVY(q)=FnmVY(q)−KV⋅FnmVZ(q) FnmVZ(q)=FnmVZ(q)+KV⋅FnmVY(q)
$\vec{R^{Y}}$	≤0	FnmVX(q)=FnmVX(q)−KV⋅FnmVZ(q) FnmVY(q)=FnmVY(q) FnmVZ(q)=FnmVZ(q)+KV⋅FnmVX(q)
	>0	FnmVX(q)=FnmVX(q)+KV⋅FnmVZ(q) FnmVY(q)=FnmVY(q) FnmVZ(q)=FnmVZ(q)−KV⋅FnmVX(q)
$\vec{R^{Z}}$	≤0	FnmVX(q)=FnmVX(q)+KV⋅FnmVY(q) FnmVY(q)=FnmVY(q)−KV⋅FnmVX(q) FnmVZ(q)=FnmVZ(q)
	>0	FnmVX(q)=FnmVX(q)−KV⋅FnmVY(q) FnmVY(q)=FnmVY(q)+KV⋅FnmVX(q) FnmVZ(q)=FnmVZ(q)

**Table 3:** Technical data of the proposed scenario.

Variable		Value	Unit
Workspace size (*x*, *y*, *z*)		(2.2, 3.8, 2.0)	(m)
Time of execution		21	(s)
Target initial position (*x*, *y*, *z*)		(1.2, 2.6, 1.2)	(m)
Target trajectory (as a function of time “*t*”)	x	0.5 sin(*t*/3.5)	(m/s)
	y	0.8 cos(*t*/3.5)	(m/s)
	z	0.7 sin(*t*/3.5)	(m/s)
Obstacle size (*x*, *y*, *z*)		(0.4, 0.4, 0.3)	(m)
Obstacle center of mass initial position (*x, y, z*)		(1.1, 1.9, 1.25)	(m)
Obstacle trajectory (as a function of time “*t*”)	x	0.02*t*	(m/s)
	y	−0.02*t*	(m/s)
	z	0.00*t*	(m/s)
Initial coordinates of the UAV 1 (*x*, *y*, *z*)		(0.6, 0.5, 0.5)	(m)
Initial coordinates of the UAV 2 (*x*, *y*, *z*)		(1.1, 0.5, 0.5)	(m)

**Table 4:** Test conditions.

Variable	Value	Unit
Attractive field gain coefficient	1	-
Repulsive field gain coefficient	2	-
Distance of influence of the obstacle	0.6	m
Vortex field gain coefficient (when applied)	4	-
UAV speed constraint	0.3	m/s

**Table 5:** Comparison metrics.

Metric	Conventional APF		LTAPF		Proposed APF	
	UAV1	UAV2	UAV1	UAV2	UAV1	UAV2
Total distance travelled (m)	5.0797	4.9327	5.1585	5.2856	5.3627	5.1027
Final target approach (m)	0.4475	0.3532	0.4089	0.3038	0.3238	0.2843
Mean target distance (m)	0.8762	0.9280	1.0378	0.8066	0.8729	0.8590
Collision	Yes	Yes	Yes	No	No	No

**Table 6:** Test results for UAV1.

Quad/Test	Axis	Max. Error (m)	Min. Error (m)	IAE
	*x*	0.396	0.000	2.516
UAV 1–Test 1	*y*	0.511	0.003	3.812
	*z*	0.379	0.000	2.937
	*x*	0.370	0.000	2.473
UAV 1–Test 2	*y*	0.435	0.001	3.772
	*z*	0.381	0.000	2.870
	*x*	0.408	0.001	2.530
UAV 1–Test 3	*y*	0.493	0.002	3.780
	*z*	0.345	0.000	2.878

**Table 7:** Test results for UAV2.

Quad/Test	Axis	Max. Error (m)	Min. Error (m)	IAE
	*x*	0.388	0.000	2.458
UAV 2–Test 1	*y*	0.470	0.002	3.635
	*z*	0.348	0.000	2.408
	*x*	0.354	0.001	2.542
UAV 2–Test 2	*y*	0.477	0.000	3.683
	*z*	0.294	0.000	2.442
	*x*	0.350	0.000	2.605
UAV 2–Test 3	*y*	0.456	0.000	3.846
	*z*	0.339	0.001	2.600

## Data Availability

Data available on request from the authors.

## Supplementary Materials

We are providing three videos showing three-dimension animations that allow visualizing the path generated by each method. The following videos are available online at https://youtu.be/jCZUijiUYF8, Video S1: Path generated by the conventional APF algorithm as proposed by Khatib, 1986; https://youtu.be/BgtQWOGpuTA, Video S2: Path generated by LTAPF as presented by Di et al, 2020; https://youtu.be/NeWscoIZ4Co, Video S3: Path generated by the modified APF algorithm proposed in this work.
